# Active learning–based reinforcement in nursing education: a cluster-randomized controlled trial using Roper, Logan and Tierney's life model

**DOI:** 10.1590/1980-220X-REEUSP-2026-0022en

**Published:** 2026-09-07

**Authors:** Nadiye Barış Eren, Bahar Çiftçi

**Affiliations:** 1 Tarsus University, Faculty of Health Sciences, Department of Nursing, Tarsus, Türkiye.; 2 Atatürk University, Faculty of Nursing, Department of Fundamentals of Nursing, Erzurum, Türkiye.; 3 HGF Agro, Erzurum, Türkiye.

**Keywords:** Education, Nursing, Students, Nursing, Learning, Educational Technology, Motivation., Educação em Enfermagem, Estudantes de Enfermagem, Aprendizagem, Tecnologia Educacional, Motivação.

## Abstract

**Objective:**

To examine the effect of puzzle-based reinforcement on nursing students’ academic achievement and motivation toward instructional materials.

**Method:**

To conduct a cluster-randomized controlled trial with 70 first-year nursing students allocated to intervention and control groups. Both groups received a 2-hour interactive lecture on Roper, Logan, and Tierney’s Life Model. The intervention group completed a one-week puzzle-based reinforcement program, while the control group received no additional intervention. Data were collected using achievement tests and the Instructional Material Motivation Scale and analyzed using paired-samples t-tests, independent-samples t-tests, and regression analysis.

**Results:**

Knowledge scores increased in the intervention group, whereas knowledge scores decreased in the control group. Between-group comparisons and regression analysis showed that puzzle-based reinforcement was associated with higher post-test knowledge scores and relatively higher motivation subscale scores compared with the control group (p < 0.01).

**Conclusion:**

The findings suggest that puzzle-based reinforcement may be a valuable instructional approach, associated with positive learning outcomes and relatively higher motivation toward instructional materials in nursing education.

**Trial Registration:**

ClinicalTrials.gov: NCT07090850.

## RESUMO

**Objetivo:**

Examinar o efeito do reforço baseado em quebra-cabeças sobre o desempenho acadêmico e a motivação dos estudantes de enfermagem em relação aos materiais instrucionais.

**Método:**

Ensaio clínico randomizado por conglomerados realizado com 70 estudantes do primeiro ano de enfermagem, alocados nos grupos intervenção e controle. Ambos os grupos participaram de uma aula interativa de duas horas sobre o Modelo de Vida de Roper, Logan e Tierney. O grupo de intervenção participou de um programa de reforço baseado em quebra-cabeças durante uma semana, enquanto o grupo controle não recebeu intervenção adicional. Os dados foram coletados por meio de testes de conhecimento e da Escala de Motivação para Materiais Instrucionais e analisados por meio de testes t pareados, testes t para amostras independentes e análise de regressão.

**Resultados:**

Os escores de conhecimento aumentaram no grupo de intervenção, enquanto diminuíram no grupo de controle. As comparações entre grupos e a análise de regressão mostraram que o reforço baseado em quebra-cabeças esteve associado a maiores escores pós-teste de conhecimento e a escores relativamente mais elevados nas subescalas de motivação em comparação com o grupo controle (p < 0,01).

**Conclusão:**

Os resultados sugerem que o reforço baseado em quebra-cabeças pode constituir uma estratégia instrucional valiosa, associada a melhores resultados de aprendizagem e a níveis relativamente mais elevados de motivação em relação aos materiais instrucionais na educação em enfermagem.

## INTRODUCTION

Education is a process in which science and art are intertwined with its conceptual-theoretical dimension, and practical skills and interventions for unique human beings are taught by integrating them with international ethics and values^(1)^. In this context, education is a challenging experience for students. Therefore, using different methods and techniques in undergraduate education is recommended. Traditional education often relies heavily on passive, lecture-based methods, which have been shown to reduce student engagement, limit long-term knowledge retention, and inadequately support the development of critical thinking and clinical decision-making skills^(2)^. Studies have identified low motivation, difficulty applying theoretical knowledge in clinical settings, and limited diversity in teaching strategies as ongoing challenges in curricula^(3,4)^. In the literature, serious games^(5)^, robots^(6)^, virtual reality^(7)^, standardized patients^(8)^, web-based education^(9)^, video-based^(10)^, blended learning^(11)^, flipped classrooms^(12,13)^, simulation^(9,14)^, scenario-based learning^(15)^, peer education^(16,17)^, and puzzles^(18,19,20,21,22)^. Puzzle-based learning aligns with the principles of active learning and is supported by constructivist learning theory, which emphasizes that learners construct their own understanding through engagement and problem-solving. Puzzles encourage cognitive processing, critical thinking, and knowledge retention by requiring students to connect concepts. This approach fosters deeper learning by promoting active involvement rather than passive information reception^(18)^. This study aims to address this gap by investigating whether puzzles can enhance both academic achievement and student motivation.

Evidence on puzzle-based learning in health professions education remains limited; however, interest in puzzles as an active learning strategy has increased^(18,19,20,21,22)^. A study by Zamani et al.^(22)^ found that students’ knowledge levels increased with the use of puzzles in education and remained high across repeated tests, indicating retention of learning. In the same study, students who used the puzzle technique reported higher satisfaction levels than those in the control group. In a study by Shawahna and Jaber^(18)^ on the pharmacological treatment of epilepsy among students, it was reported that the puzzle increased students’ learning. In another study conducted by Köse Tosunöz and Deniz Doğan^(19)^ on students, it was reported that students’ knowledge levels increased. A study by Qutieshat et al.^(21)^ on dentistry students revealed that students found the puzzles interesting, meaningful, and successful. With puzzles, students are encouraged to think and gain satisfaction by answering questions. This satisfaction also increases their motivation and academic success. Therefore, using the puzzle technique in teaching contributes to education. Puzzle-based learning may improve knowledge retention and motivation by engaging students in active recall and application; however, evidence of its effectiveness in teaching comprehensive nursing care models, such as Roper, Logan, and Tierney’s Life Model, remains limited.

As healthcare systems become increasingly complex, innovative, evidence-based approaches are necessary to enhance the preparedness of future nurses. This research offers practical implications for enhancing curriculum design and teaching practices in education. This initiative aims to increase students’ participation and motivation in the course.

### Research Questions

1. Does the use of puzzles in education improve students’ academic achievement?
2. Does the use of puzzles enhance students’ motivation towards instructional materials?

## METHOD

### Type of Study

This study used a cluster-randomized pretest–posttest controlled experimental design.

### Population and Sample of the Study

The first-year cohort was organised into three teaching branches. To minimise contamination, randomisation was conducted at the branch (cluster) level. The study population consisted of 270 students. Within the scope of accreditation, each branch attended the course in the same classroom, and the same lecturer taught the subject using the same method. To prevent contamination, a cluster-randomized design was used. Among the three branches, two were selected by lot, and one branch was assigned to the intervention group and the other to the control group. Two of the three branches (A and C) were randomly selected and then randomly allocated to intervention (C) and control (A) by drawing lots. Using G*Power 3.1.9.7, the minimum sample size was calculated as 34 students per group (α = 0.05, power = 0.80). To account for potential data loss, 70 students were included (35 in the intervention group, 35 in the control group). Because the study used a two-cluster design, the sample size calculation was based on individual-level assumptions and should be interpreted with caution.

### Inclusion Criteria

First-year nursing students aged ≥18 years who were taking the Basic Concepts and Philosophy course for the first time and had not previously received education on Roper, Logan and Tierney’s Life Model.

### Exclusion Criteria

Students who did not complete the puzzles in the intervention group or reported access to the puzzle materials in the control group.

### Data Collection Tools

Personal Information Form, Achievement Test 1, Achievement Test 2, Instructional Material Motivation Scale (IMTS), and Puzzle Booklet were used to collect the data.

*Personal Information Form:* Prepared by the researchers in line with the literature, this form included questions on students’ demographic characteristics, such as age, gender, and socioeconomic level.

***Instructional Material Motivation Scale (IMTS):*** The Turkish version of the IMTS includes 33 items across four dimensions (attention, relevance, confidence, satisfaction). The original scale developed by Keller contains 36 items^(23)^. Cronbach’s Alpha value for the whole scale is .96. Cronbach’s Alpha values of the scale dimensions: attention dimension .89, relevance dimension .81, confidence dimension .90, and satisfaction dimension, .92. In this study, the Cronbach’s Alpha value of the IMTS was found to be 0.92. The scale is a 5-point Likert-type scale, scored as Very True (5), True (4), Moderately True (3), Somewhat True (2), and Not True (1)^(24)^. The highest score on the whole scale is 165, and the lowest is 33. The highest score in the attention dimension is 50, and the lowest is 10. The highest score in the relevance dimension is 40, and the lowest is 8. The highest score for the confidence dimension is 45, and the lowest is 9. The highest score in the satisfaction dimension is 30, and the lowest is 6. An increase in the score on the scale indicates greater motivation towards the teaching material. The total score obtained from the scale was expressed as very low (33.00–37.00), low (37.01–54.00), medium (54.001–143.00), high (144.01–160.99), and very high (161.00–165). The IMTS is an adapted tool and has been previously published. It is not unique to this research, and its original and adapted sources are cited in the manuscript.

***Achievement Test 1 and Achievement Test 2:*** The researchers prepared multiple-choice questions after a literature review. For each test, they developed items/questions related to Roper, Logan, and Tierney’s Life Model for Life Activities. An initial pool of 50 multiple-choice items was developed based on the literature and learning objectives. Two parallel forms were created (Achievement Test 1 and 2), each containing 25 items with comparable content coverage and difficulty.

The study participants were taken to the classroom, and Achievement Test 1 was administered as a pretest. The evaluation of the achievement tests was based on a 100-point scale. For this purpose, the students’ total achievement scores were calculated as 4 times the number of correct answers. The results of Achievement Test 1 and Achievement Test 2 were evaluated by giving 4 points for each correct answer and 0 points for each incorrect answer. The highest score students could get from the achievement tests was 100, and the lowest was 0.

Achievement Test 1 and Achievement Test 2 included the topic Roper, Logan, and Tierney’s Life Model for Life Activities. The topics were distributed equally. The achievement tests were not used to determine students’ performance or assessment grades. The questions were prepared for the first time and were specific to this research. In addition, these questions were not used in the midterm or final exams; this information was explained to the students. For the achievement tests, expert opinions were obtained from five faculty members from the Department of Fundamentals of Nursing. The questions were finalized based on the experts’ views. Achievement Test 1 and Achievement Test 2 were developed explicitly for this study. In addition, both tests were reviewed by experts to ensure content equivalence and comparable difficulty levels. Although formal psychometric equivalence testing was not conducted, both forms were developed based on the same learning objectives and content framework to ensure comparability. Both tests were designed to assess the same learning outcomes and cognitive levels, ensuring consistency in content coverage and difficulty. The items were structured to discriminate between different levels of student performance. This approach aimed to minimise measurement bias and ensure that differences in scores reflected actual learning rather than variations in test characteristics.

***Puzzle Booklet: Design, Development, and Implementation:*** This study employed a Puzzle Booklet as an innovative learning tool to reinforce the theoretical content of Roper, Logan, and Tierney’s Life Model for Life Activities. The development of the Puzzle Booklet began with the creation of an item pool of approximately 200 questions aligned with the model’s core concepts and learning objectives. From this item pool, 100 items were selected and organized into a booklet containing **10 puzzle sheets**, with approximately 10 items per sheet**.** Across these sheets, the content was delivered through **multiple puzzle formats** (e.g., matching, word search, crossword, and clinical case-based items), created using an online puzzle generator. For example, in a crossword activity, students were asked to define key life activities such as elimination or communication, while in matching activities, they were required to pair specific interventions with the corresponding life activities.

The rationale for selecting these puzzle types was to promote essential cognitive skills, including recall**,** critical thinking**,** problem-solving**,** and the application of theoretical knowledge to clinical practice. The puzzles were designed explicitly for first-year students, with attention to balancing cognitive load—ensuring they were challenging enough to engage students without being overwhelming.

To validate the content and ensure alignment with course objectives, the Puzzle Booklet was reviewed by **five faculty experts**. This expert review process helped ensure that the puzzles were conceptually accurate, appropriately challenging, and relevant to the course material. Content validity was established through expert review. Given that the Puzzle Booklet was used as an instructional reinforcement tool rather than an outcome measure, formal psychometric testing was not conducted.

In terms of classroom implementation, the Puzzle Booklet was integrated into the broader lesson plan following a 2-hour interactive lecture on the Life Model. Students in the intervention group were instructed to complete the puzzles individually, both during class time and as follow-up assignments. Instructors monitored participation, provided feedback, and ensured that the puzzles were used as intended. The puzzle activities were designed to function as a formative learning tool, encouraging students to engage in self-directed problem-solving, consolidate their understanding, and reflect on the theoretical content.

Overall, the Puzzle Booklet was not only a means to reinforce course content but also a structured approach to active learning and cognitive engagement. By aligning the puzzles with learning objectives, balancing cognitive demands, and integrating them into the course flow, the study aimed to ensure methodological rigour and facilitate the broader application of puzzle-based learning in education.

### Data Collection

**Figure 1:**
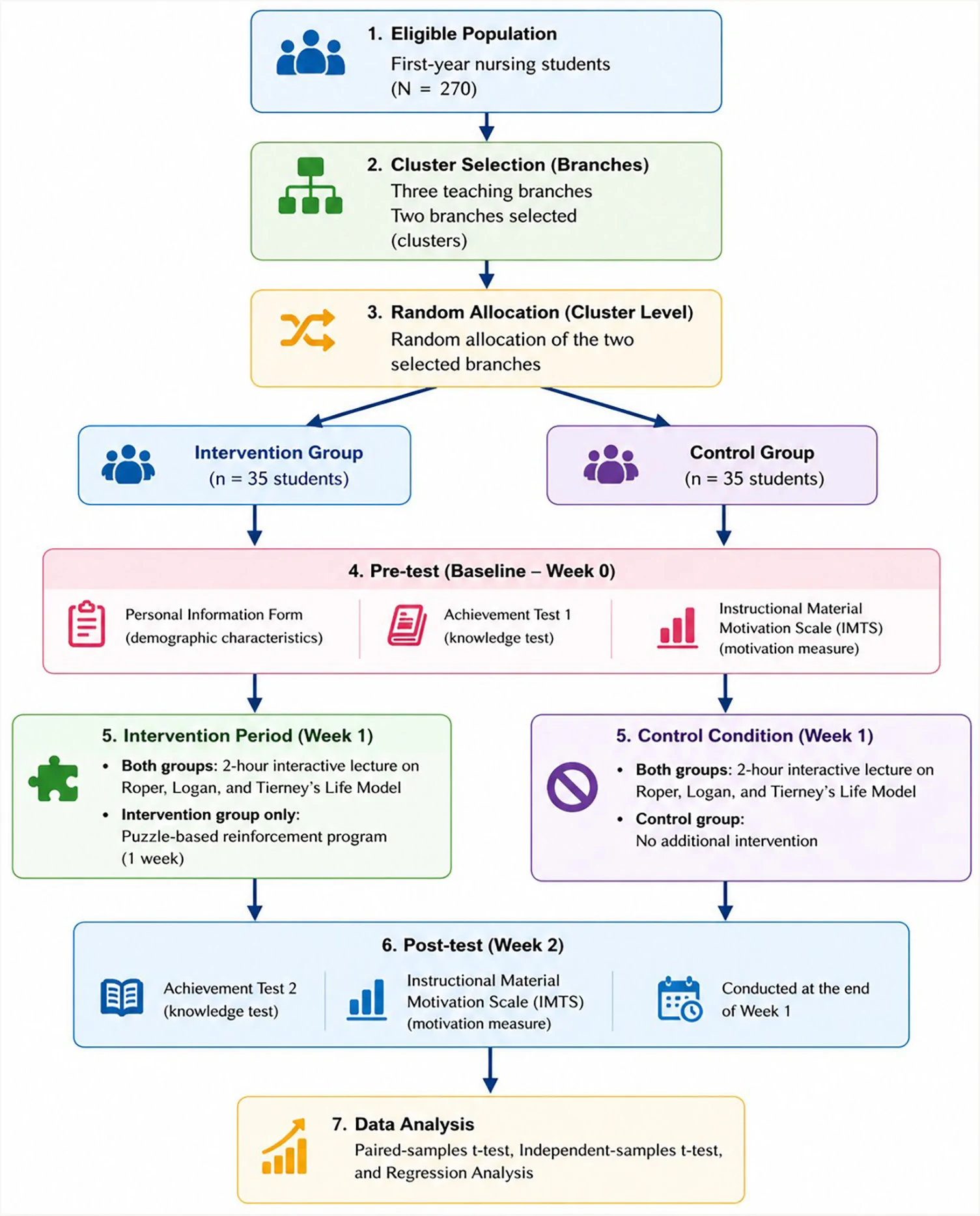
Flowchart of the data collection process and study procedure.

Data were collected during the Spring 2024 semester. The data collection process and study procedure are illustrated in Figure 1.

In the first stage, all students were informed about the research and provided consent. The Personal Information Form was completed by students who provided informed consent and agreed to participate in the study under observation in the classroom environment. Then, the Achievement Test 1 and the IMTS were administered to all students as a pretest. Roper Logan and Tierney’s Life Model for Life Activities elective course was among the courses offered to first-year students. The course is 2 hours long and theoretical. In the second stage, Roper, Logan, and Tierney’s Life Model for Life Activities was taught interactively to all students (experimental and control) by the same instructors for 2 hours for 1 week.

The puzzle booklet previously created for this study was used in the third stage. During the lesson, the booklet containing 10 puzzle sheets was distributed to students at the end of each hour and collected at the end of the lesson. In addition, the puzzle booklet was distributed to the students to be repeated later, and all of them were collected at the end of 1 week. The collected booklets were checked, deficiencies and mistakes were noted, and these mistakes were discussed individually with the students, and the correct ones were shown. In addition, students in the intervention group were instructed not to share the puzzle materials with peers in the control group to minimise contamination.

In the Control Group (Branch A), routine lecturing continued without intervention in the fourth stage. In the fifth stage, Achievement Test 2 was administered to the students in both the intervention and control groups 1 week after the training, and IMTS was administered as a post-test.

### Data Evaluation

IBM SPSS Statistics version 22.0 was used to evaluate the data. Frequency and percentage were used to determine the distribution of sociodemographic characteristics. Chi-square analysis compared the experimental and control groups on descriptive traits. Skewness and Kurtosis values were used to determine whether the measurements were usually distributed. Paired-samples t-tests were used for within-group comparisons, and independent-samples t-tests were used for between-group comparisons. Cohen’s d value was used to determine the effect size. Regression analysis was performed to assess the effect of puzzle application on the mean scores. A statistical significance value of 0.05 was accepted. Because only two clusters were included, multilevel or cluster-adjusted analyses were not feasible. Therefore, analyses were conducted at the individual level, and the results should be interpreted with caution due to the potential for inflated statistical significance.

### Ethical Principles of Research

The study was approved by the Atatürk University Faculty of Nursing Ethics Committee (Approval No. 2023-12/1, Date: 01 December 2023). The trial was prospectively registered at ClinicalTrials.gov (Identifier: NCT07090850). Written informed consent was obtained from all participants. Participation was voluntary, confidentiality was ensured, and the study was conducted in accordance with the Declaration of Helsinki. The researchers were also involved in the teaching process. To minimize potential bias related to authority relationships, participation in the study was entirely voluntary, and students were assured that their responses would not affect their academic evaluation. Data collection was conducted anonymously, and no identifying information was obtained. To minimize contamination, students in the intervention group were instructed not to share the puzzle materials with peers in the control group. After completion, the puzzle booklet was also provided to the control group.

It was stated that the research results would not affect any performance evaluations, midterms, or final grades, and that the control group would be given the puzzle application and the booklet in the same way after the research was completed. Students in the intervention group were reminded not to share the puzzle materials with peers in the control group during the study period to minimize contamination. At the end of the study, all students in the control group were also given the puzzle booklet to avoid ethical problems.

## RESULTS

This study was conducted with 70 first-year students enrolled in the Basic Concepts and Philosophy at Atatürk University. Participants were allocated to the intervention group (n = 35) and the control group (n = 35) within each cluster (branch). All participants completed the study, and no participants were lost to follow-up; therefore, no sample attrition occurred. The demographic characteristics of the participants are summarised in Table 1.

**Table 1:** Distribution of descriptive characteristics of students (n = 70) – Erzurum, Türkiye, 2024.

Variables		Groups				Test value and significance
		Experiment (n = 35)		Control (n = 35)		
		n	%	n	(%)	
Age	18–19	22	62.9	23	65.7	χ^2^ = 0.062, p = 0.803^a^
	20 and above	13	37.1	12	34.3
Gender	Female	27	77.1	31	88.6	χ^2^ = 1.609, p = 0.205^a^
	Male	8	22.9	4	11.4
Family structure	Nuclear	28	80.0	28	80.0	χ^2^ = 0.001, p = 1.000^a^
	Extended family	7	20.0	7	20.0
Current residence	Family home	6	17.1	14	40.0	**χ^2^ = 4.480, p = 0.034^a^**
	Dormitory	29	82.9	21	60.0
Perceived income status	Good	14	40.0	18	51.4	χ^2^ = 0.921, p = 0.337^a^
	Low	21	60.0	17	48.6
Longest place of residence	Village	9	25.7	9	25.7	χ^2^ = 0.080, p = 0.961^a^
	District	11	31.4	10	28.6
	Province	15	42.9	16	45.7
Voluntary choice of department	Yes	17	48.6	26	74.3	χ^2^ = 4.884, p = 0.027^a^
	No	18	51.4	9	25.7
Sense of professional belonging	Yes	28	80.0	29	82.9	χ^2^ = 0.094, p = 0.759^a^
	No	7	20.0	6	17.1
Chronic disease status	Yes	5	14.3	2	5.7	p = 0.428^b^
	No	30	85.7	33	94.3
Enjoyment of mobile gaming	Yes	15	42.9	13	37.1	χ^2^ = 0.238, p = 0.626^a^
	No	20	57.1	22	62.9
Daily time spent on mobile games	0	16	45.7	15	42.9	χ^2^ = 1.306, p = 0.520^a^
	1 hour	11	31.4	8	22.9
	2 hours and over	8	22.9	12	34.3
Main source of information	Mass media	23	65.7	19	54.3	χ^2^ = 0.952, p = 0.329^a^
	Other	12	34.3	16	45.7
Use of puzzles for education	Yes	13	37.1	10	28.6	χ^2^ = 0.583, p = 0.445^a^
	No	22	62.9	25	71.4
Use of mobile games for education	Yes	18	51.4	20	57.1	χ^2^ = 0.230, p = 0.631^a^
	No	17	48.6	15	42.9
Number of books read per month	1–2	23	65.7	15	42.9	χ^2^ = 3.684, p = 0.055^a^
	3 and above	12	34.3	20	57.1	

a = Pearson chi-square test;

b = Fisher’s exact test.

It was determined that 62.9% of the students in the experimental group were between the ages of 18-19, 77.1% were female, 80% had a nuclear family, 82.9% lived in a dormitory, 60% had a poor income, 42.9% lived in the province, 51.4% did not choose the department willingly, 80% felt that they belonged to the profession, 85.7% did not have chronic diseases, 57.1% did not like to play mobile games. It was determined that 45.7% of the students in the experimental group did not play any games during the day, 65.7% obtained information mostly from mass media, 62.9% did not make puzzles for education, 51.4% played mobile games for education, and 65.7% read 1–2 books per month (Table 1).

It was found that 65.7% of the students in the control group were between the ages of 18–19, 88.6% were female, 80% had a nuclear family, 60% lived in a dormitory, 51.4% had a good income, 45.7% lived in the province, 74.3% chose the department willingly, 82.9% felt that they belonged to the profession, 94.3% did not have chronic diseases, and 62.9% did not like to play mobile games. It was determined that 42.9% of the students in the control group did not play any games during the day; 54.3% obtained information mainly from the mass media; 71.4% did not make puzzles for educational purposes; 57.1% played mobile games for academic purposes; and 57.1% read three or more books per month. The results of the chi-square analysis showed that the experimental and control groups were not homogeneous with respect to the variable current place of residence. Still, they were homogeneous across other descriptive variables (Table 1).

In within-group comparisons, it was determined that there was no statistically significant difference between the pre-test and post-test mean scores of the students in the experimental group for the Attention Subscale, Relevance Subscale, Confidence Subscale, Satisfaction Subscale, and Motivation Scale for Instructional Material (p > 0.05). It was determined that the mean post-test score of the students in the experimental group was higher than the mean pre-test score, and this difference was statistically significant (p < 0.05) (Table 2).

**Table 2:** Comparison of students’ in-group and between-group mean scores – Erzurum, Türkiye, 2024.

Groups		Experiment	Control	Intergroup test value and significance^χ^
		X– ± SD	X– ± SD	
Attention	Pre-test^1^	38.61 ± 8.86	37.46 ± 6.54	t = 0.645 p = 0.521 ES = 0.154
	Post-test^2^	41.20 ± 7.76	35.43 ± 7.80	**t = 3.104 p = 0.003 ES = 0.742**
	In-group Test Value and Significance^y^	t = −1.364 p = 0.181 ES = 0.231	t = 1.576 p = 0.124 ES = 0.266
Relevance	Pre-test^1^	31.51 ± 6.76	31.60 ± 4.80	t = −0.061 p = 0.951 ES = 0.015
	Post-test^2^	32.74 ± 5.25	28.74 ± 6.81	**t = 2.750 p = 0.008 ES = 0.657**
	In-group Test Value and Significance^y^	t = −0.988 p = 0.330 ES = 0.167	**t = 2.565 p = 0.015 ES = 0.434**
Confidence	Pre-test^1^	34.74 ± 6.98	32.91 ± 5.73	t = 1.198 p = 0.235 ES = 0.286
	Post-test^2^	36.51 ± 6.42	31.29 ± 7.27	**t = 3.189 p = 0.002 ES = 0.762**
	In-group Test Value and Significance^y^	t = −1.293 p = 0.205 ES = 0.219	t = 1.391 p = 0.173 ES = 0.235
Satisfaction	Pre-test^1^	23.34 ± 5.24	22.17 ± 4.52	t = 1.001 p = 0.320 ES = 0.239
	Post-test^2^	24.48 ± 4.60	21.17 ± 5.63	**t = 2.697 p = 0.009 ES = 0.645**
	In-group Test Value and Significance^y^	t = −1.086 p = 0.285 ES = 0.184	t = 1.020 p = 0.315 ES = 0.172
Motivation Scale for Instructional Material (Total)	Pre-test^1^	128.26 ± 26.88	124.14 ± 20.17	t = 0.724 p = 0.471 ES = 0.173
	Post-test^2^	134.94 ± 23.24	116.63 ± 26.96	**t = 3.044 p = 0.003 ES = 0.728**
	In-group Test Value and Significance^y^	t = −1.277 p = 0.210 ES = 0.216	t = 1.728 p = 0.093 ES = 0.292
Knowledge Test	Pre-test^1^	67.77 ± 7.13	64.57 ± 13.96	t = 1.208 p = 0.231 ES = 0.289
	Post-test^2^	79.09 ± 17.80	37.14 ± 13.83	**t = 11.008 p = 0.001 ES = 2.631**
	In-group Test Value and Significance^y^	t = −4.219 p = 0.001 ES = 0.713	**t = 12.106 p = 0.001 ES = 2.046**	

x: Independent-samples t-test;

y: Paired-samples t-test; ES: Cohen’s d.

It was determined that there was no statistically significant difference between the pre-test and post-test mean scores of the students in the control group for the Attention Subscale, Relevance Subscale, Confidence Subscale, Satisfaction Subscale, and Motivation Scale for Instructional Material (p > 0.05). It was determined that the mean pre-test score of the students in the control group was higher than the mean post-test score, and this difference was statistically significant (p < 0.05). It was determined that the mean pre-test score on the Knowledge Test for the control group was higher than the mean post-test score, and this difference was statistically significant (p < 0.05) (Table 2).

When the comparisons between the groups were analysed, it was found that there was no statistically significant difference in the mean pre-test scores for the Attention Subscale, Relevance Subscale, Confidence Subscale, Satisfaction Subscale, and Motivation Scale for Instructional Material and Knowledge Test between the experimental and control groups (p > 0.05). The experimental group had a higher mean post-test score than the control group on the Attention Subscale, and this difference was statistically significant (p < 0.05). It was determined that the experimental group’s mean post-test score was higher than that of the control group, and the difference was statistically significant (p < 0.05). The experimental group’s mean posttest score on the Confidence Subscale was higher than that of the control group, and the difference was statistically significant (p < 0.05). It was determined that the experimental group had a higher mean posttest score of Satisfaction Subscale than the control group, and this difference was statistically significant (p < 0.05). It was determined that the experimental group’s mean posttest score was higher than the control group’s on the Motivation Scale for Instructional Material, and this difference was statistically significant (p < 0.05). It was determined that the experimental group’s mean post-test score was higher than the control group’s on the Knowledge Test, and this difference was statistically significant (p < 0.05).

Table 3 presents the results of the Regression Analysis with Representative Variable conducted to determine the effect of puzzle application on the dependent variables. It was found that the six models were statistically significant (p < 0.05). When the regression coefficients were examined, it was found that the puzzle application had a significant effect on Attention Subscale (β = 0.352, p = 0.003), Relevance Subscale (β = 0.316, p = 0.008), Confidence Subscale (β = 0.361, p = 0.002), Satisfaction Subscale (β = 0.311, p = 0.009), Motivation Scale for Instructional Material (β = 0.346, p = 0.003) and Knowledge Test (β = 0.800, p = 0.001) (Table 3).

**Table 3:** Regression analysis results regarding the effect of puzzle application on score averages – Erzurum, Türkiye, 2024.

Dependent variable	Model	Variables	B	S.Error	β	*t*	*p*
Attention	1	Constant	35.429	1.315		26.946	.001
		Puzzle Application – Experiment	5.771	1.859	.352	3.104	.003
**R = 0.352, R2 = 0.124, F = 9.635, p = 0.003**							
Relevance	1	Constant	28.743	1.028		27.948	.001
		Puzzle Application – Experiment	4.000	1.454	.316	2.750	.008
**R = 0.316, R2 = 0.100, F = 7.563, p = 0.008**							
Confidence	1	Constant	31.286	1.160		26.982	.001
		Puzzle Application – Experiment	5.229	1.640	.361	3.189	.002
**R = 0.361, R2 = 0.130, F = 10.167, p = 0.002**							
Satisfaction	1	Constant	21.171	.869		24.360	.001
		Puzzle Application – Experiment	3.314	1.229	.311	2.697	.009
**R = 0.311, R2 = 0.097, F = 7.271, p = 0.009**							
Motivation Scale for Instructional Material	1	Constant	116.629	4.254		27.415	.001
		Puzzle Application – Experiment	18.314	6.016	.346	3.044	.003
**R = 0.346, R2 = 0.120, F = 9.266, p = 0.003**							
Knowledge Test	1	Constant	37.143	2.694		13.786	.001
		Puzzle Application – Experiment	41.943	3.810	.800	11.008	.001
**R = 0.800, R2 = 0.641, F = 121.170, p = 0.001**							

## DISCUSSION

In this cluster-randomized controlled study conducted in an undergraduate nursing course at a state university, knowledge scores increased in the experimental group, whereas they decreased in the control group. This finding suggests that knowledge retention in the control group declined over time. The substantial decrease in knowledge scores observed in the control group may be explained by several factors. First, the absence of reinforcement following the initial lecture may have contributed to reduced retention over time. Second, although the achievement tests were designed as parallel forms with comparable content and difficulty, minor variations between test forms cannot be completely excluded. Third, uncontrolled factors such as individual study habits, engagement levels, and external learning conditions during the follow-up period may have influenced the results. These factors should be considered when interpreting the observed decline in the control group. It should be noted that baseline differences between groups, particularly in terms of residence status and voluntary choice of the nursing programme, may have influenced the outcomes. These variables may reflect differences in study conditions and levels of academic engagement and should be considered as potential confounding factors when interpreting the intervention effects. Given that only two clusters were included, such baseline imbalances are more likely and represent a limitation of the study design. A study conducted by Demir Acar and Çaylak Altun^(4)^ among students found that visual learning styles were most common, followed by auditory and physical learning styles. A study found that students found puzzles as a teaching tool interesting, promoted active participation, enhanced information retention, and supported dynamic self-evaluation^(4)^. These findings are consistent with our results showing higher knowledge scores following puzzle-based reinforcement. It can be said that using puzzles in education as a teaching technique is practical and may support information retention. Students generally perceive puzzles as enjoyable and meaningful learning activities, and studies have reported high satisfaction and engagement with crossword-based learning^(19,21,22)^. A study by Aydin and Ince^(25)^ found that using puzzles in education improved students’ psychomotor skills and increased information retention. The study by Köse Tosunöz and Deniz Doğan^(19)^ on the use of puzzles in education found that students’ knowledge levels in the experimental group after the training were higher than those in the control group. A study conducted by Zamani et al.^(22)^ reported that students’ knowledge levels increased when puzzles were used in education^(22)^. Knowledge levels were high across repeated tests, indicating learning retention. A study by Torres et al.^(20)^ found that graduate students who engaged in puzzle-solving were more successful than those who did not. The meta-analysis by Ozkan and Uslusoy^(26)^ on the use of the puzzle technique in education found that students’ academic achievement, skills, and attitudes were positively affected by the use of puzzles. Unlike our study, in a study by Sumanasekera et al.^(27)^, although 60% to 66% of the students reported that puzzles helped them remember the concepts, no significant difference in students’ knowledge scores was found.

It was determined that the students’ motivation regarding the instructional material in the experimental and control groups was moderate. Similarly, the study by Çetinkaya Uslusoy et al.^(3)^ on metaverse-based education and academic achievement and learning motivation in students found that students’ motivation was moderate after the education. On the other hand, after the puzzle intervention, the experimental group showed higher motivation than the control group. This result suggests that the puzzle technique may have supported student participation and engagement and may have contributed to relatively higher motivation toward the instructional material. The literature includes studies on the effects of various teaching methods and techniques on students’ motivation to learn. Ongor and Uslusoy^(28)^ found that students’ academic achievement and motivation increased through multimedia-based distance learning. A study by Çetinkaya Uslusoy et al.^(3)^ found that metaverse-based education increased students’ motivation to learn and academic achievement. Likewise, Bozkurt et al.^(29)^ demonstrated that ARCS-based nursing education effectively enhanced students’ learning motivation. Our findings suggest that, although no significant within-group change was observed in the intervention group, post-test motivation scores were higher compared to the control group. Therefore, the observed difference may reflect a relative advantage between groups rather than a clear increase over time within the intervention group. These findings should be interpreted with caution when considering the direct effect of the intervention on motivation. On the other hand, unlike our study, Aras and Çiftçi^(30)^ found that the question-and-answer and Kahoot methods did not significantly affect students’ motivation. This between-group difference may reflect the motivational benefits of active, student-centred reinforcement strategies such as crossword puzzles, which have been associated with learner engagement in nursing and health professions education^(19,21,22)^. Future studies using cluster-randomized designs should consider incorporating the design effect in sample size calculations and employing analytical approaches that account for clustering, such as linear mixed models or generalized estimating equations (GEE), to enhance methodological robustness.

## LIMITATIONS

This study was conducted with a specific sample of first year students from a single university, which may limit the generalizability of the findings to other programs or educational contexts. Additionally, the potential for interaction between students in the intervention and control groups, despite efforts to minimize contamination, is acknowledged as a limitation that could have influenced the study outcomes. Additionally, randomization was conducted at the cluster level with only two clusters, and the analyses were performed at the individual level, which may have limited control of cluster effects. Another important limitation is the use of a cluster-randomized design with only two clusters. Because statistical analyses were conducted at the individual level without adjustment for clustering, the findings may be overestimated in statistical significance. Therefore, the results should be interpreted with caution regarding internal validity.

## CONCLUSION

This study examined the effect of puzzles in education on students’ academic achievement and motivation. Significant improvements were observed in knowledge levels, and higher post-test motivation scores were identified compared to the control group. These increases were not observed in the control group. The results suggest that puzzle-based learning may be a useful teaching approach and is associated with positive learning outcomes. While the results are promising, it is important to interpret these findings within the context of this study. Further research is needed to explore the effectiveness of puzzle-based learning across diverse educational settings, subjects, and student populations to ensure the generalizability of these results. In this context, the puzzle technique may have contributed to increased student interest in the lesson, enhanced information retention, and greater enjoyment of learning. While the findings are promising, they should be interpreted with caution given the study’s methodological limitations. Further research with larger samples and more robust cluster-randomized designs is needed to confirm these results and strengthen the evidence base.

Based on the results of this study, the use of puzzles in education was associated with higher knowledge scores and relatively higher motivation levels compared with the control group. In line with these results, it is recommended that interactive, student-centered teaching techniques, such as puzzles, be used more widely in faculties, especially in theoretical courses. Thus, students’ participation in the course may increase, learning may become more engaging, and information retention may be supported. In addition, integrating puzzles and other game-based learning tools into the development of educational materials may increase students’ motivation. In addition to traditional teaching methods, it is essential to employ a range of teaching techniques that cater to students’ diverse learning styles. Methods such as simulation, scenario-based learning, and flipped classrooms may further support students’ learning processes. Finally, more research is needed to examine the effects of teaching techniques, such as puzzles, across different subjects and student groups. In this way, the effectiveness of teaching methods can be evaluated from a broader perspective, and innovative approaches can be adopted more in education.

## Data Availability

The datasets generated and/or analysed during the current study are available within the article.
