# Supplementary material for: Active learning–based reinforcement in nursing education: a cluster-randomized controlled trial using Roper, Logan and Tierney's life model
Source: Rev Esc Enferm USP. 2026 Sep 7;60:e20260022. doi: 10.1590/1980-220X-REEUSP-2026-0022en (PMC13551602; doi:10.1590/1980-220X-REEUSP-2026-0022en)
Supplement: Translation

# Translation: Refuerzo basado en el aprendizaje activo en la educación en enfermería: un ensayo controlado aleatorizado por conglomerados utilizando el modelo de vida de Roper, Logan y Tierney

**DOI:** 10.1590/1980-220X-REEUSP-2026-0022es

**Authors:** Nadiye Barış Eren, Bahar Çiftçi

**Affiliations:** 1 Tarsus University, Faculty of Health Sciences, Department of Nursing, Tarsus, Türkiye.; 2 Atatürk University, Faculty of Nursing, Department of Fundamentals of Nursing, Erzurum, Türkiye.; 3 HGF Agro, Erzurum, Türkiye.

**Keywords:** Educación en Enfermería, Estudiantes de Enfermería, Aprendizaje, Tecnología Educacional, Motivación.

## Abstract

**Objetivo:**

Examinar el efecto del refuerzo basado en rompecabezas sobre el rendimiento académico y la motivación de los estudiantes de enfermería hacia los materiales didácticos.

**Método:**

Ensayo controlado aleatorizado por conglomerados realizado con 70 estudiantes de primer año de enfermería asignados a los grupos de intervención y control. Ambos grupos recibieron una clase interactiva de dos horas sobre el Modelo de Vida de Roper, Logan y Tierney. El grupo de intervención completó un programa de refuerzo basado en rompecabezas durante una semana, mientras que el grupo control no recibió ninguna intervención adicional. Los datos se recopilaron mediante pruebas de conocimientos y la Escala de Motivación hacia los Materiales Didácticos y se analizaron mediante pruebas t pareadas, pruebas t para muestras independientes y análisis de regresión.

**Resultados:**

Las puntuaciones de conocimiento aumentaron en el grupo de intervención, mientras que disminuyeron en el grupo control. Las comparaciones entre grupos y el análisis de regresión mostraron que el refuerzo basado en rompecabezas se asoció con puntuaciones más altas en la prueba posterior de conocimientos y con puntuaciones relativamente superiores en las subescalas de motivación en comparación con el grupo control (p < 0,01).

**Conclusión:**

Los hallazgos sugieren que el refuerzo basado en rompecabezas puede constituir una estrategia educativa valiosa, asociada con mejores resultados de aprendizaje y niveles relativamente superiores de motivación hacia los materiales didácticos en la educación en enfermería.

**Registro del Ensayo:**

ClinicalTrials.gov: NCT07090850.

## INTRODUCCIÓN

La educación es un proceso en el que la ciencia y el arte se entrelazan con su dimensión conceptual-teórica, y en el que se enseñan habilidades prácticas e intervenciones para seres humanos únicos, integrándolas con la ética y los valores internacionales^(1)^. En este contexto, la educación es una experiencia desafiante para los estudiantes. Por lo tanto, se recomienda utilizar diferentes métodos y técnicas en la educación de pregrado. La educación tradicional suele depender en gran medida de métodos pasivos basados en clases magistrales, los cuales han demostrado reducir la participación de los estudiantes, limitar la retención de conocimientos a largo plazo y no apoyar adecuadamente el desarrollo del pensamiento crítico y las habilidades para la toma de decisiones clínicas^(2)^. Diversos estudios han identificado la baja motivación, la dificultad para aplicar los conocimientos teóricos en entornos clínicos y la diversidad limitada en las estrategias de enseñanza como desafíos recurrentes en los planes de estudio^(3,4)^. En la literatura, se han identificado los juegos serios^(5)^, los robots^(6)^, la realidad virtual^(7)^, los pacientes estandarizados^(8)^, la educación basada en la web^(9)^, la enseñanza basada en videos^(10)^, el aprendizaje combinado^(11)^, las aulas invertidas^(12,13)^, la simulación^(9,14)^, el aprendizaje basado en escenarios^(15)^, la educación entre pares^(16,17)^ y los rompecabezas^(18,19,20,21,22)^. El aprendizaje basado en rompecabezas se alinea con los principios del aprendizaje activo y está respaldado por la teoría constructivista del aprendizaje, la cual enfatiza que los alumnos construyen su propio entendimiento a través de la participación y la resolución de problemas. Los rompecabezas fomentan el procesamiento cognitivo, el pensamiento crítico y la retención de conocimientos al exigir que los estudiantes conecten conceptos. Este enfoque fomenta un aprendizaje más profundo al promover la participación activa en lugar de la recepción pasiva de información^(18)^. Este estudio tiene como objetivo abordar esta brecha al investigar si los rompecabezas pueden mejorar tanto el desempeño académico como la motivación de los estudiantes.

La evidencia sobre el aprendizaje basado en rompecabezas en la formación de profesiones de la salud sigue siendo limitada; sin embargo, ha aumentado el interés en los rompecabezas como estrategia de aprendizaje activo^(18,19,20,21,22)^. Un estudio de Zamani et al.^(22)^ encontró que los niveles de conocimiento de los estudiantes aumentaron con el uso de rompecabezas en la educación y se mantuvieron altos en pruebas repetidas, lo que indica la retención del aprendizaje. En el mismo estudio, los estudiantes que utilizaron la técnica de los rompecabezas reportaron niveles de satisfacción más altos que los del grupo de control. En un estudio de Shawahna y Jaber^(18)^ sobre el tratamiento farmacológico de la epilepsia entre estudiantes, se informó que los rompecabezas mejoraron el aprendizaje de los estudiantes. En otro estudio realizado por Köse Tosunöz y Deniz Doğan^(19)^ con estudiantes, se informó que los niveles de conocimiento de los estudiantes aumentaron^(19)^. Un estudio de Qutieshat et al.^(21)^ con estudiantes de odontología reveló que los estudiantes consideraban que los rompecabezas eran interesantes, significativos y exitosos. Con los rompecabezas, se anima a los estudiantes a pensar y a obtener satisfacción al responder preguntas. Esta satisfacción también aumenta su motivación y su éxito académico. Por lo tanto, el uso de la técnica de los rompecabezas en la enseñanza contribuye a la educación. El aprendizaje basado en rompecabezas puede mejorar la retención de conocimientos y la motivación al involucrar a los estudiantes en la recuperación activa y la aplicación de la información; sin embargo, la evidencia de su efectividad en la enseñanza de modelos integrales de atención de enfermería, como el Modelo de Vida de Roper, Logan y Tierney, sigue siendo limitada.

A medida que los sistemas de salud se vuelven cada vez más complejos, se requieren enfoques innovadores y basados en la evidencia para mejorar la preparación de los futuros enfermeros. Esta investigación ofrece implicaciones prácticas para mejorar el diseño curricular y las prácticas docentes en la educación. Esta iniciativa tiene como objetivo aumentar la participación y la motivación de los estudiantes en el curso.

### Preguntas de Investigación

1. ¿El uso de rompecabezas en la educación mejora el desempeño académico de los estudiantes?
2. ¿El uso de rompecabezas aumenta la motivación de los estudiantes hacia los materiales didácticos?

## MÉTODO

### Tipo de Estudio

Este estudio utilizó un diseño experimental controlado de preprueba y posprueba con aleatorización por conglomerados.

### Población y Muestra del Estudio

La cohorte de primer año se organizó en tres ramas de enseñanza. Para minimizar la contaminación, la aleatorización se llevó a cabo a nivel de rama (grupo). La población del estudio estuvo compuesta por 270 estudiantes. En el marco de la acreditación, cada rama asistió al curso en el mismo salón de clases, y el mismo profesor impartió la materia utilizando el mismo método. Para evitar la contaminación, se utilizó un diseño aleatorizado por grupos. De las tres secciones, se seleccionaron dos por sorteo, asignándose una sección al grupo de intervención y la otra al grupo de control. Dos de las tres secciones (A y C) fueron seleccionadas al azar y luego asignadas aleatoriamente a intervención (C) y control (A) mediante sorteo. Utilizando G*Power 3.1.9.7, se calculó que el tamaño mínimo de la muestra era de 34 estudiantes por grupo (α = 0,05, potencia = 0,80). Para tener en cuenta la posible pérdida de datos, se incluyeron 70 estudiantes (35 en el grupo de intervención y 35 en el grupo de control). Dado que el estudio utilizó un diseño de dos grupos, el cálculo del tamaño de la muestra se basó en supuestos a nivel individual y debe interpretarse con precaución.

### Criterios de Inclusión

Estudiantes de primer año de enfermería de 18 años o más que cursaban por primera vez la asignatura “Conceptos básicos y filosofía” y que no habían recibido previamente formación sobre el Modelo de Vida de Roper, Logan y Tierney.

### Criterios de Exclusión

Estudiantes que no completaron los rompecabezas en el grupo de intervención o que informaron haber tenido acceso a los materiales de los rompecabezas en el grupo de control.

### Herramientas Para la Recolección de Datos

Para recolectar los datos se utilizaron el Formulario de Información Personal, la Prueba de Desempeño 1, la Prueba de Desempeño 2, la Escala de Motivación hacia el Material Didáctico (*Instructional Material Motivation Scale* - IMTS) y el Folleto de Rompecabezas.

*Formulario de información personal:* Elaborado por los investigadores de acuerdo con la literatura, este formulario incluía preguntas sobre las características demográficas de los estudiantes, tales como edad, género y nivel socioeconómico.

***Escala de Motivación Hacia el Material Didáctico (IMTS):*** La versión turca de la IMTS incluye 33 ítems distribuidos en cuatro dimensiones (atención, relevancia, confianza y satisfacción). La escala original desarrollada por Keller contiene 36 ítems^(23)^. El valor del alfa de Cronbach para toda la escala es de 0,96. Los valores del alfa de Cronbach de las dimensiones de la escala son: dimensión de atención 0,89; dimensión de relevancia 0,81; dimensión de confianza 0,90; y dimensión de satisfacción 0,92. En este estudio, se determinó que el valor del alfa de Cronbach de la IMTS era de 0,92. La escala es de tipo Likert de 5 puntos, con las siguientes puntuaciones: “Muy cierto” (5), “Cierto” (4), “Moderadamente cierto” (3), “Algo cierto” (2) y “No cierto” (1)^(24)^. La puntuación máxima en toda la escala es 165 y la mínima es 33. La puntuación máxima en la dimensión de atención es 50 y la mínima es 10. La puntuación más alta en la dimensión de relevancia es 40, y la más baja es 8. La puntuación más alta en la dimensión de confianza es 45, y la más baja es 9. La puntuación más alta en la dimensión de satisfacción es 30, y la más baja es 6. Un aumento en la puntuación de la escala indica una mayor motivación hacia el material didáctico. La puntuación total obtenida en la escala se clasificó como muy baja (33,00-37,00), baja (37,01-54,00), media (54,001-143,00), alta (144,01-160,99) y muy alta (161,00-165). El IMTS es una herramienta adaptada que ya ha sido publicada anteriormente. No es exclusiva de esta investigación, y tanto su fuente original como la adaptada se citan en el manuscrito.

***Prueba de Desempeño 1 y Prueba de Desempeño 2:*** Los investigadores elaboraron preguntas de opción múltiple tras una revisión de la literatura. Para cada prueba, desarrollaron ítems o preguntas relacionados con el Modelo de Vida para las Actividades de la Vida de Roper, Logan y Tierney. Se elaboró un conjunto inicial de 50 ítems de opción múltiple basado en la literatura y los objetivos de aprendizaje. Se crearon dos formas paralelas (Prueba de Desempeño 1 y 2), cada una con 25 ítems con cobertura de contenido y dificultad comparables.

Se llevó a los participantes del estudio al salón de clases y se les aplicó la Prueba de Desempeño 1 como prueba previa. La evaluación de las pruebas de desempeño se basó en una escala de 100 puntos. Para ello, las puntuaciones totales de desempeño de los estudiantes se calcularon multiplicando por cuatro el número de respuestas correctas. Los resultados de la Prueba de Desempeño 1 y la Prueba de Desempeño 2 se evaluaron otorgando 4 puntos por cada respuesta correcta y 0 puntos por cada respuesta incorrecta. La puntuación más alta que los estudiantes podían obtener en las pruebas de desempeño era 100, y la más baja, 0.

La Prueba de Desempeño 1 y la Prueba de Desempeño 2 incluían el tema “Modelo de vida para las actividades de la vida diaria” de Roper, Logan y Tierney. Los temas se distribuyeron de manera equitativa. Las pruebas de desempeño no se utilizaron para determinar el desempeño de los estudiantes ni para asignar calificaciones de evaluación. Las preguntas se elaboraron por primera vez y eran específicas para esta investigación. Además, estas preguntas no se utilizaron en los exámenes parciales ni finales; esto se explicó a los estudiantes. Para las pruebas de desempeño, se recabaron opiniones de cinco miembros del cuerpo docente del Departamento de Fundamentos de Enfermería. Las preguntas se definieron definitivamente con base en las opiniones de los expertos. La Prueba de Desempeño 1 y la Prueba de Desempeño 2 se desarrollaron específicamente para este estudio. Además, ambas pruebas fueron revisadas por expertos para garantizar la equivalencia de contenido y niveles de dificultad comparables. Aunque no se realizaron pruebas formales de equivalencia psicométrica, ambas versiones se desarrollaron con base en los mismos objetivos de aprendizaje y el mismo marco de contenido para garantizar la comparabilidad. Ambas pruebas se diseñaron para evaluar los mismos resultados de aprendizaje y niveles cognitivos, asegurando la consistencia en la cobertura del contenido y la dificultad. Los ítems se estructuraron para discriminar entre diferentes niveles de desempeño de los estudiantes. Este enfoque tuvo como objetivo minimizar el sesgo de medición y garantizar que las diferencias en las calificaciones reflejaran el aprendizaje real y no variaciones en las características de la prueba.

***Cuaderno de Rompecabezas: Diseño, Desarrollo e Implementación:*** Este estudio utilizó un cuaderno de rompecabezas como herramienta de aprendizaje innovadora para reforzar el contenido teórico del Modelo de Vida para las Actividades de la Vida de Roper, Logan y Tierney. El desarrollo del folleto de rompecabezas comenzó con la creación de un banco de ítems de aproximadamente 200 preguntas alineadas con los conceptos centrales y los objetivos de aprendizaje del modelo. De este banco de ítems, se seleccionaron 100 ítems y se organizaron en un folleto que contiene 10 hojas de rompecabezas, con aproximadamente 10 ítems por hoja. En todas estas hojas, el contenido se presentó a través de múltiples formatos de rompecabezas (por ejemplo, emparejamiento, búsqueda de palabras, crucigramas y preguntas basadas en casos clínicos), creados con un generador de rompecabezas en línea. Por ejemplo, en una actividad de crucigrama, se les pidió a los estudiantes que definieran actividades clave de la vida, como la eliminación o la comunicación, mientras que en las actividades de emparejamiento, debían asociar intervenciones específicas con las actividades de la vida correspondientes.

La razón para seleccionar estos tipos de rompecabezas fue promover habilidades cognitivas esenciales, entre ellas la memoria, el pensamiento crítico, la resolución de problemas, y la aplicación del conocimiento teórico a la práctica clínica. Los rompecabezas se diseñaron específicamente para estudiantes de primer año, prestando atención al equilibrio de la carga cognitiva—asegurándose de que fueran lo suficientemente desafiantes como para captar el interés de los estudiantes sin resultar abrumadores.

Para validar el contenido y garantizar la alineación con los objetivos del curso, el Folleto de Acertijos fue revisado por cinco expertos del cuerpo docente. Este proceso de revisión por expertos ayudó a garantizar que los rompecabezas fueran conceptualmente precisos, presentaran un desafío adecuado y fueran relevantes para el material del curso. La validez de contenido se estableció mediante la revisión de los expertos. Dado que el Cuadernillo de rompecabezas se utilizó como una herramienta de refuerzo educativo y no como una medida de resultados, no se realizaron pruebas psicométricas formales.

En cuanto a la implementación en el salón de clases, el Cuadernillo de rompecabezas se integró en el plan de lecciones más amplio después de una clase interactiva de dos horas sobre el Modelo de Vida. A los estudiantes del grupo de intervención se les indicó que completaran los rompecabezas de manera individual, tanto durante el horario de clase como en tareas de seguimiento. Los instructores monitorearon la participación, brindaron retroalimentación y se aseguraron de que los rompecabezas se utilizaran según lo previsto. Las actividades de los rompecabezas se diseñaron para funcionar como una herramienta de aprendizaje formativo, que alentara a los estudiantes a participar en la resolución autónoma de problemas, a consolidar su comprensión y a reflexionar sobre el contenido teórico.

En general, el Cuadernillo de rompecabezas no solo fue un medio para reforzar el contenido del curso, sino también un enfoque estructurado para el aprendizaje activo y la participación cognitiva. Al alinear los rompecabezas con los objetivos de aprendizaje, equilibrar las exigencias cognitivas e integrarlos en el flujo del curso, el estudio buscó garantizar el rigor metodológico y facilitar una aplicación más amplia del aprendizaje basado en rompecabezas en la educación.

### Recolección de Datos

Los datos se recolectaron durante el semestre de primavera de 2024. El proceso de recolección de datos y el procedimiento del estudio se ilustran en la Figura 1.

**Figura 1:** Diagrama de flujo del proceso de recolección de datos y del procedimiento del estudio.

En la primera etapa, se informó a todos los estudiantes sobre la investigación y estos dieron su consentimiento. Los estudiantes que dieron su consentimiento informado y aceptaron participar en el estudio bajo observación en el entorno del salón de clases completaron el Formulario de información personal. Luego, se administraron la Prueba de desempeño 1 y el IMTS a todos los estudiantes como prueba previa. El curso optativo “Modelo de Vida para las Actividades de la Vida” de Roper, Logan y Tierney se encontraba entre los cursos ofrecidos a los estudiantes de primer año. El curso tiene una duración de 2 horas y es teórico. En la segunda etapa, el “Modelo de Vida para las Actividades de la Vida” de Roper, Logan y Tierney se impartió de manera interactiva a todos los estudiantes (del grupo experimental y del grupo de control) por parte de los mismos instructores durante 2 horas a lo largo de una semana.

En la tercera etapa se utilizó el folleto de rompecabezas creado previamente para este estudio. Durante la clase, el folleto que contenía 10 hojas de rompecabezas se distribuyó a los estudiantes al final de cada hora y se recolectó al terminar la clase. Además, se entregó a los estudiantes el folleto de rompecabezas para que lo repitieran más tarde, y todos se recolectaron al final de una semana. Se revisaron los folletos recolectados, se anotaron las deficiencias y los errores, se discutieron estos errores individualmente con los estudiantes y se les mostraron las respuestas correctas.

Además, se indicó a los estudiantes del grupo de intervención que no compartieran los materiales de los rompecabezas con sus compañeros del grupo de control para minimizar la contaminación.

En el grupo de control (Sección A), se continuó con las clases magistrales habituales sin intervención en la cuarta etapa. En la quinta etapa, se aplicó la Prueba de Logro 2 a los estudiantes tanto del grupo de intervención como del de control una semana después de la capacitación, y se aplicó el IMTS como post-test.

### Evaluación de Datos

Se utilizó IBM SPSS Statistics versión 22.0 para evaluar los datos. Se emplearon la frecuencia y el porcentaje para determinar la distribución de las características sociodemográficas. El análisis de chi-cuadrado comparó los grupos experimental y de control en cuanto a rasgos descriptivos. Se utilizaron los valores de asimetría y curtosis para determinar si las mediciones seguían una distribución normal. Se utilizaron pruebas t para muestras emparejadas para las comparaciones intragrupales, y pruebas t para muestras independientes para las comparaciones intergrupales. Se utilizó el valor d de Cohen para determinar el tamaño del efecto. Se realizó un análisis de regresión para evaluar el efecto de la aplicación de rompecabezas en los puntajes promedio. Se aceptó un nivel de significancia estadística de 0,05. Dado que solo se incluyeron dos grupos, no fue posible realizar análisis multinivel o ajustados por grupos. Por lo tanto, los análisis se llevaron a cabo a nivel individual, y los resultados deben interpretarse con precaución debido a la posibilidad de que la significancia estadística esté inflada.

### Principios Éticos de la Investigación

El estudio fue aprobado por el Comité de Ética de la Facultad de Enfermería de la Universidad Atatürk (N.º de aprobación 2023-12/1, fecha: 1 de diciembre de 2023). El ensayo se registró prospectivamente en ClinicalTrials.gov (identificador: NCT07090850). Se obtuvo el consentimiento informado por escrito de todos los participantes. La participación fue voluntaria, se garantizó la confidencialidad y el estudio se llevó a cabo de conformidad con la Declaración de Helsinki. Los investigadores también participaban en el proceso de enseñanza. Para minimizar cualquier posible sesgo relacionado con las relaciones de autoridad, la participación en el estudio fue totalmente voluntaria y se aseguró a los estudiantes que sus respuestas no afectarían su evaluación académica. La recolección de datos se realizó de forma anónima y no se obtuvo información que permitiera identificar a los participantes. Para minimizar la contaminación, se indicó a los estudiantes del grupo de intervención que no compartieran los materiales del juego de rompecabezas con sus compañeros del grupo de control. Una vez finalizado el estudio, también se entregó el folleto de rompecabezas al grupo de control.

Se les informó que los resultados de la investigación no afectarían ninguna evaluación de desempeño, exámenes parciales ni calificaciones finales, y que el grupo de control recibiría la aplicación de rompecabezas y el folleto de la misma manera una vez finalizada la investigación. Se les recordó a los estudiantes del grupo de intervención que no compartieran los materiales de los rompecabezas con sus compañeros del grupo de control durante el período de estudio para minimizar la contaminación. Al final del estudio, también se entregó el folleto de rompecabezas a todos los estudiantes del grupo de control para evitar problemas éticos.

## RESULTADOS

Este estudio se llevó a cabo con 70 estudiantes de primer año matriculados en la asignatura de Conceptos Básicos y Filosofía de la Universidad de Atatürk. Los participantes fueron asignados al grupo de intervención (n = 35) y al grupo de control (n = 35) dentro de cada grupo (rama). Todos los participantes completaron el estudio y no se perdió a ningún participante durante el seguimiento; por lo tanto, no se produjo ningún desgaste de la muestra. Las características demográficas de los participantes se resumen en la Tabla 1.

**Tabla 1:** Distribución de las características descriptivas de los estudiantes (n = 70) – Erzurum, Türkiye, 2024.

Variables		Grupos				Valor de la prueba y significación
		Experimental (n = 35)		Control (n = 35)		
		n	%	n	(%)	
Edad	18-19	22	62,9	23	65,7	χ^2^ = 0,062, p = 0,803^a^
	20 y más	13	37,1	12	34,3
Género	Mujer	27	77,1	31	88,6	χ^2^ = 1,609, p = 0,205^a^
	Hombres	8	22,9	4	11,4
Estructura familiar	Nuclear	28	80,0	28	80,0	χ^2^ = 0,001, p = 1,000^a^
	Familia extensa	7	20,0	7	20,0
Residencia actual	Vivienda familiar	6	17,1	14	40,0	**χ^2^ = 4,480, p = 0,034^a^**
	Residencia	29	82,9	21	60,0
Nivel de ingresos percibido	Buena	14	40,0	18	51,4	χ^2^ = 0,921, p = 0,337^a^
	Bajo	21	60,0	17	48,6
Lugar de residencia más prolongado	Pueblo	9	25,7	9	25,7	χ^2^ = 0,080, p = 0,961^a^
	Distrito	11	31,4	10	28,6
	Provincia	15	42,9	16	45,7
Elección voluntaria del departamento	Sí	17	48,6	26	74,3	χ^2^ = 4,884, p = 0,027^a^
	No	18	51,4	9	25,7
Sentido de pertenencia profesional	Sí	28	80,0	29	82,9	χ^2^ = 0,094, p = 0,759^a^
	No	7	20,0	6	17,1
Estado Estado de la enfermedad	Sí	5	14,3	2	5,7	p = 0,428^b^
	No	30	85,7	33	94,3
Disfrute de los juegos para móvil	Sí	15	42,9	13	37,1	χ^2^ = 0,238, p = 0,626^a^
	No	20	57,1	22	62,9
Tiempo diario dedicado a los juegos para móvil	0	16	45,7	15	42,9	χ^2^ = 1,306, p = 0,520^a^
	1 hora	11	31,4	8	22,9
	2 horas o más	8	22,9	12	34,3
Principal fuente de información	Medios de comunicación	23	65,7	19	54,3	χ^2^ = 0,952, p = 0,329^a^
	Otros	12	34,3	16	45,7
Uso de los rompecabezas con fines educativos	Sí	13	37,1	10	28,6	χ^2^ = 0,583, p = 0,445^a^
	No	22	62,9	25	71,4
Uso de los juegos para móviles con fines educativos	Sí	18	51,4	20	57,1	χ^2^ = 0,230, p = 0,631^a^
	No	17	48,6	15	42,9
Número de libros leídos al mes	1–2	23	65,7	15	42,9	χ^2^ = 3,684, p = 0,055^a^
	3 y más	12	34,3	20	57,1	

a = prueba de chi-cuadrado de Pearson;

b = prueba exacta de Fisher.

Se determinó que el 62,9% de los estudiantes del grupo experimental tenían entre 18 y 19 años, el 77,1% eran mujeres, el 80% provenía de una familia nuclear, el 82,9% vivía en una residencia estudiantil, el 60% tenía bajos ingresos, el 42,9% vivía en el campo, el 51,4% no había elegido la carrera por voluntad propia, el 80% sentía que pertenecía a la profesión, el 85,7% no padecía enfermedades crónicas y al 57,1% no le gustaba jugar juegos en el celular.

Se determinó que el 45,7% de los estudiantes del grupo experimental no jugaba a ningún juego durante el día, el 65,7% obtenía información principalmente de los medios de comunicación, el 62,9% no resolvía rompecabezas con fines educativos, el 51,4% jugaba a juegos en el celular con fines educativos y el 65,7% leía de 1 a 2 libros al mes (Tabla 1).

Se observó que el 65,7% de los estudiantes del grupo de control tenía entre 18 y 19 años, el 88,6% eran mujeres, el 80% pertenecía a una familia nuclear, el 60% vivía en una residencia estudiantil, el 51,4% contaba con buenos ingresos, el 45,7% vivía en una provincia, el 74,3% había elegido la carrera por voluntad propia, el 82,9% sentía que pertenecía a la profesión, el 94,3% no padecía enfermedades crónicas y al 62,9% no le gustaba jugar juegos en el celular. Se determinó que el 42,9% de los estudiantes del grupo de control no jugaba ningún juego durante el día; el 54,3% obtenía información principalmente de los medios de comunicación; el 71,4% no resolvía rompecabezas con fines educativos; el 57,1% jugaba juegos en el celular con fines académicos; y el 57,1% leía tres o más libros al mes. Los resultados del análisis de chi-cuadrado mostraron que los grupos experimental y de control no eran homogéneos con respecto a la variable “lugar de residencia actual”. Sin embargo, sí eran homogéneos en cuanto a otras variables descriptivas (Tabla 1).

En las comparaciones intragrupales, se determinó que no existía una diferencia estadísticamente significativa entre las puntuaciones medias de la prueba previa y la prueba posterior de los alumnos del grupo experimental en las subescalas de Atención, Relevancia, Confianza y Satisfacción, así como en la Escala de Motivación respecto al material didáctico (p > 0,05). Se determinó que la puntuación media de la prueba posterior de los alumnos del grupo experimental era superior a la puntuación media de la prueba previa, y que esta diferencia era estadísticamente significativa (p < 0,05) (Tabla 2).

**Tabla 2:** Comparación de las puntuaciones medias intragrupales e intergrupales de los alumnos – Erzurum, Türkiye, 2024.

Grupos		Experimental	Control	Valor y significación de la prueba intergrupal^χ^
		X– ± DE	X– ± DE	
Atención	Prueba previa^1^	38,61 ± 8,86	37,46 ± 6,54	t = 0,645 p = 0,521 ES = 0,154
	Prueba posterior^2^	41,20 ± 7,76	35,43 ± 7,80	**t = 3,104 p = 0,003 ES = 0,742**
	Valor y significación de la prueba intragrupal^y^	t = −1,364 p = 0,181 ES = 0,231	t = 1,576 p = 0,124 ES = 0,266
Relevancia	Prueba previa^1^	31,51 ± 6,76	31,60 ± 4,80	t = −0,061 p = 0,951 ES = 0,015
	Prueba posterior^2^	32,74 ± 5,25	28,74 ± 6,81	**t = 2,750 p = 0,008 ES = 0,657**
	Valor de la prueba intragrupal y significación^y^	t = −0,988 p = 0,330 ES = 0,167	**t = 2,565 p = 0,015 ES = 0,434**
Nivel de confianza	Prueba previa^1^	34,74 ± 6,98	32,91 ± 5,73	t = 1,198 p = 0,235 ES = 0,286
	Prueba posterior^2^	36,51 ± 6,42	31,29 ± 7,27	**t = 3,189 p = 0,002 ES = 0,762**
	Valor de la prueba intragrupal y significación^y^	t = −1,293 p = 0,205 ES = 0,219	t = 1,391 p = 0,173 ES = 0,235
Satisfacción	Prueba previa^1^	23,34 ± 5,24	22,17 ± 4,52	t = 1,001 p = 0,320 ES = 0,239
	Prueba posterior^2^	24,48 ± 4,60	21,17 ± 5,63	**t = 2,697 p = 0,009 ES = 0,645**
	Valor de la prueba intragrupal y significación^y^	t = −1,086 p = 0,285 ES = 0,184	t = 1,020 p = 0,315 ES = 0,172
Escala de motivación para el material didáctico (total)	Prueba previa^1^	128,26 ± 26,88	124,14 ± 20,17	t = 0,724 p = 0,471 ES = 0,173
	Prueba posterior^2^	134,94 ± 23,24	116,63 ± 26,96	**t = 3,044 p = 0,003 ES = 0,728**
	Valor de la prueba intragrupal y significación^y^	t = −1,277 p = 0,210 ES = 0,216	t = 1,728 p = 0,093 ES = 0,292
Prueba de conocimientos	Prueba previa^1^	67,77 ± 7,13	64,57 ± 13,96	t = 1,208 p = 0,231 ES = 0,289
	Prueba posterior^2^	79,09 ± 17,80	37,14 ± 13,83	**t = 11,008 p = 0,001 ES = 2,631**
	Valor y significación de la prueba intragrupaly	t = −4,219 p = 0,001 ES = 0,713	**t = 12,106 p = 0,001 ES = 2,046**	

x: prueba t para muestras independientes;

y: prueba t para muestras emparejadas; ES: d de Cohen.

Se determinó que no existía ninguna diferencia estadísticamente significativa entre las puntuaciones medias de la prueba previa y la prueba posterior de los alumnos del grupo de control en las subescalas de Atención, Relevancia, Confianza y Satisfacción, así como en la Escala de Motivación hacia el Material Didáctico (p > 0,05). Se determinó que la puntuación media en la prueba previa de los alumnos del grupo de control era superior a la puntuación media en la prueba posterior, y que esta diferencia era estadísticamente significativa (p < 0,05). Se determinó que la puntuación media en la prueba previa de conocimientos del grupo de control era superior a la puntuación media en la prueba posterior, y que esta diferencia era estadísticamente significativa (p < 0,05) (Tabla 2).

Al analizar las comparaciones entre los grupos, se observó que no existía ninguna diferencia estadísticamente significativa en las puntuaciones medias previas a la prueba en las subescalas de Atención, Relevancia, Confianza y Satisfacción, así como en la Escala de Motivación respecto al material didáctico y en la prueba de conocimientos, entre el grupo experimental y el grupo de control (p > 0,05). El grupo experimental obtuvo una puntuación media en la prueba posterior más alta que el grupo de control en la subescala de atención, y esta diferencia fue estadísticamente significativa (p < 0,05). Se determinó que la puntuación media en la prueba posterior del grupo experimental era superior a la del grupo de control, y que la diferencia era estadísticamente significativa (p < 0,05). La puntuación media en la prueba posterior del grupo experimental en la subescala de “Confianza” fue superior a la del grupo de control, y la diferencia fue estadísticamente significativa (p < 0,05). Se determinó que el grupo experimental obtuvo una puntuación media en la prueba posterior de la subescala de “Satisfacción” superior a la del grupo de control, y esta diferencia fue estadísticamente significativa (p < 0,05). Se determinó que la puntuación media del grupo experimental en la prueba posterior era superior a la del grupo de control en la “Escala de motivación hacia el material didáctico”, y esta diferencia fue estadísticamente significativa (p < 0,05). Se determinó que la puntuación media del grupo experimental en la prueba posterior era superior a la del grupo de control en la “Prueba de conocimientos”, y esta diferencia fue estadísticamente significativa (p < 0,05).

La Tabla 3 presenta los resultados del análisis de regresión con variable representativa realizado para determinar el efecto de la aplicación de rompecabezas sobre las variables dependientes. Se observó que los seis modelos eran estadísticamente significativos (p < 0,05). Al examinar los coeficientes de regresión, se observó que la aplicación de rompecabezas tenía un efecto significativo en la subescala de atención (β = 0,352; p = 0,003), la subescala de relevancia (β = 0,316; p = 0,008), la subescala de confianza (β = 0,361; p = 0,002), la subescala de satisfacción (β = 0,311; p = 0,009), la escala de motivación respecto al material didáctico (β = 0,346; p = 0,003) y la prueba de conocimientos (β = 0,800; p = 0,001) (Tabla 3).

**Tabla 3:** Resultados del análisis de regresión sobre el efecto de la aplicación de rompecabezas en las puntuaciones medias – Erzurum, Türkiye, 2024.

Variable dependiente	Modelo	Variables	B	Error estándar	β	*t*	*p*
Atención	1	Constante	35,429	1,315		26,946	0,001
		Rompecabezas Aplicación – Experimento	5,771	1,859	.352	3,104	0,003
**R = 0,352, R^2^ = 0,124, F = 9,635, p = 0,003**							
Relevancia	1	Constante	28,743	1,028		27,948	0,001
		Rompecabezas Aplicación – Experimento	4,000	1,454	.316	2.750	0,008
**R = 0,316, R^2^ = 0,100, F = 7,563, p = 0,008**							
Confianza	1	Constante	31,286	1,160		26,982	,001
		Aplicación de rompecabezas – Experimento	5,229	1,640	.361	3,189	0,002
**R = 0,361, R^2^ = 0,130, F = 10,167, p = 0,002**							
Satisfacción	1	Constante	21,171	,869		24,360	,001
		Aplicación de rompecabezas – Experimento	3,314	1,229	.311	2,697	0,009
**R = 0,311, R^2^ = 0,097, F = 7,271, p = 0,009**							
Escala de motivación “ “ para material didáctico	1	Constante	116,629	4,254		27,415	,001
		Aplicación de rompecabezas –Experimento 18,314	6,016	.346	3,044	0,003	
**R = 0,346, R^2^ = 0,120, F = 9,266, p = 0,003**							
Prueba de conocimientos	1	Constante	37,143	2,694		13,786	,001
		Aplicación de rompecabezas – Experimento	41,943	3,810	,800	11,008	0,001
**R = 0,800, R^2^ = 0,641, F = 121,170, p = 0,001**							

## DISCUSIÓN

En este estudio controlado aleatorizado por conglomerados, realizado en un curso de pregrado de enfermería en una universidad estatal, las puntuaciones de conocimiento aumentaron en el grupo experimental, mientras que disminuyeron en el grupo de control. Este hallazgo sugiere que la retención de conocimientos en el grupo de control disminuyó con el tiempo. La disminución sustancial en las puntuaciones de conocimiento observada en el grupo de control puede explicarse por varios factores. En primer lugar, la ausencia de refuerzo después de la clase inicial pudo haber contribuido a una menor retención con el paso del tiempo. En segundo lugar, aunque las pruebas de desempeño se diseñaron como formas paralelas con contenido y dificultad comparables, no se pueden descartar por completo variaciones menores entre las distintas formas de la prueba. En tercer lugar, factores no controlados, como los hábitos de estudio individuales, los niveles de participación y las condiciones externas de aprendizaje durante el período de seguimiento, pueden haber influido en los resultados. Estos factores deben tenerse en cuenta al interpretar la disminución observada en el grupo de control. Cabe señalar que las diferencias iniciales entre los grupos, particularmente en cuanto a la situación de residencia y la elección voluntaria del programa de enfermería, pueden haber influido en los resultados. Estas variables pueden reflejar diferencias en las condiciones de estudio y los niveles de compromiso académico, y deben considerarse como posibles factores de confusión al interpretar los efectos de la intervención. Dado que solo se incluyeron dos grupos, es más probable que existan tales desequilibrios iniciales, lo que representa una limitación del diseño del estudio. Un estudio realizado por Demir Acar y Çaylak Altun^(4)^ entre estudiantes reveló que los estilos de aprendizaje visual eran los más comunes, seguidos por los estilos auditivos y físicos. Otro estudio encontró que los estudiantes consideraban que los rompecabezas eran una herramienta didáctica interesante, que promovía la participación activa, mejoraba la retención de información y favorecía la autoevaluación dinámica^(4)^. Estos hallazgos concuerdan con nuestros resultados, que muestran puntuaciones de conocimiento más altas tras el refuerzo basado en rompecabezas. Se puede afirmar que el uso de los rompecabezas en la educación como técnica de enseñanza es práctico y puede favorecer la retención de información. Los estudiantes suelen percibir los rompecabezas como actividades de aprendizaje divertidas y significativas, y los estudios han reportado altos niveles de satisfacción y compromiso con el aprendizaje basado en crucigramas^(19,21,22)^. Un estudio de Aydin e Ince^(25)^ reveló que el uso de rompecabezas en la educación mejoró las habilidades psicomotoras de los estudiantes y aumentó la retención de información. El estudio de Köse Tosunöz y Deniz Doğan^(19)^ sobre el uso de rompecabezas en la educación reveló que los niveles de conocimiento de los estudiantes del grupo experimental después de la capacitación fueron más altos que los del grupo de control. Un estudio realizado por Zamani et al.^(22)^ informó que los niveles de conocimiento de los estudiantes aumentaron cuando se utilizaron rompecabezas en la educación. Los niveles de conocimiento se mantuvieron altos en pruebas repetidas, lo que indica la retención del aprendizaje. Un estudio de Torres et al.^(20)^ reveló que los estudiantes de posgrado que se dedicaron a resolver rompecabezas tuvieron más éxito que aquellos que no lo hicieron. El metaanálisis de Ozkan y Uslusoy^(26)^ sobre el uso de la técnica de los rompecabezas en la educación encontró que el desempeño académico, las habilidades y las actitudes de los estudiantes se vieron afectados positivamente por el uso de los rompecabezas. A diferencia de nuestro estudio, en un trabajo de Sumanasekera et al.^(27)^, aunque entre el 60% y el 66% de los estudiantes informaron que los rompecabezas les ayudaron a recordar los conceptos, no se encontró una diferencia significativa en las calificaciones de conocimiento de los estudiantes.

Se determinó que la motivación de los estudiantes respecto al material didáctico, tanto en el grupo experimental como en el de control, fue moderada. De manera similar, el estudio de Çetinkaya Uslusoy et al.^(3)^ sobre la educación basada en el metaverso y el desempeño académico y la motivación para el aprendizaje en los estudiantes reveló que la motivación de los estudiantes fue moderada después de la educación. Por otro lado, tras la intervención con rompecabezas, el grupo experimental mostró una mayor motivación que el grupo de control. Este resultado sugiere que la técnica de los rompecabezas pudo haber favorecido la participación y el compromiso de los estudiantes y pudo haber contribuido a una motivación relativamente mayor hacia el material didáctico. La literatura incluye estudios sobre los efectos de diversos métodos y técnicas de enseñanza en la motivación de los estudiantes para aprender. Ongor y Uslusoy^(28)^ encontraron que el desempeño académico y la motivación de los estudiantes aumentaron a través del aprendizaje a distancia basado en multimedia. Un estudio de Çetinkaya Uslusoy et al.^(3)^ reveló que la educación basada en el metaverso aumentó la motivación de los estudiantes para aprender y su desempeño académico. Del mismo modo, Bozkurt et al.^(29)^ demostraron que la educación en enfermería basada en el modelo ARCS mejoró de manera efectiva la motivación de los estudiantes para aprender. Nuestros hallazgos sugieren que, aunque no se observó un cambio significativo dentro del grupo de intervención, las puntuaciones de motivación en el post-test fueron más altas en comparación con el grupo de control. Por lo tanto, la diferencia observada podría reflejar una ventaja relativa entre los grupos más que un claro aumento a lo largo del tiempo dentro del grupo de intervención. Estos hallazgos deben interpretarse con cautela al considerar el efecto directo de la intervención sobre la motivación. Por otro lado, a diferencia de nuestro estudio, Aras y Çiftçi^(30)^ encontraron que los métodos de preguntas y respuestas y Kahoot no afectaron significativamente la motivación de los estudiantes. Esta diferencia entre grupos podría reflejar los beneficios motivacionales de las estrategias de refuerzo activas y centradas en el estudiante, como los crucigramas, que se han asociado con la participación de los estudiantes en la educación de enfermería y profesiones de la salud^(19,21,22)^. Los estudios futuros que utilicen diseños aleatorizados por conglomerados deberían considerar la incorporación del efecto de diseño en los cálculos del tamaño de la muestra y el empleo de enfoques analíticos que tengan en cuenta la agrupación, como los modelos lineales mixtos o las ecuaciones de estimación generalizadas (GEE), para mejorar la solidez metodológica.

## LIMITACIONES

Este estudio se llevó a cabo con una muestra específica de estudiantes de primer año de una sola universidad, lo que podría limitar la generalizabilidad de los hallazgos a otros programas o contextos educativos. Además, se reconoce como una limitación la posibilidad de interacción entre los estudiantes de los grupos de intervención y de control, a pesar de los esfuerzos por minimizar la contaminación, lo cual podría haber influido en los resultados del estudio. Además, la aleatorización se llevó a cabo a nivel de conglomerado con solo dos conglomerados, y los análisis se realizaron a nivel individual, lo que pudo haber limitado el control de los efectos de conglomerado. Otra limitación importante es el uso de un diseño aleatorizado por conglomerados con solo dos conglomerados. Dado que los análisis estadísticos se realizaron a nivel individual sin ajuste por agrupamiento, la significación estadística de los hallazgos podría estar sobreestimada. Por lo tanto, los resultados deben interpretarse con cautela en lo que respecta a la validez interna.

## CONCLUSIÓN

Este estudio examinó el efecto de los rompecabezas en la educación sobre el desempeño académico y la motivación de los estudiantes. Se observaron mejoras significativas en los niveles de conocimiento y se identificaron puntuaciones de motivación más altas en la prueba post-test en comparación con el grupo de control. Estos aumentos no se observaron en el grupo de control. Los resultados sugieren que el aprendizaje basado en rompecabezas puede ser un enfoque didáctico útil y está asociado con resultados de aprendizaje positivos. Si bien los resultados son prometedores, es importante interpretar estos hallazgos dentro del contexto de este estudio. Se necesitan más investigaciones para explorar la efectividad del aprendizaje basado en rompecabezas en diversos entornos educativos, materias y poblaciones de estudiantes, a fin de garantizar la generalizabilidad de estos resultados. En este contexto, la técnica de los rompecabezas pudo haber contribuido a un mayor interés de los estudiantes en la lección, a una mejor retención de la información y a un mayor disfrute del aprendizaje. Si bien los hallazgos son prometedores, deben interpretarse con cautela dadas las limitaciones metodológicas del estudio. Se necesitan más investigaciones con muestras más grandes y diseños aleatorios por conglomerados más sólidos para confirmar estos resultados y fortalecer la base de evidencia.

Según los resultados de este estudio, el uso de rompecabezas en la educación se asoció con puntuaciones de conocimiento más altas y niveles de motivación relativamente más elevados en comparación con el grupo de control. En consonancia con estos resultados, se recomienda que las técnicas de enseñanza interactivas y centradas en el estudiante, como los rompecabezas, se utilicen más ampliamente en las facultades, especialmente en los cursos teóricos. De este modo, podría aumentar la participación de los estudiantes en el curso, el aprendizaje podría volverse más atractivo y se podría favorecer la retención de la información. Además, la integración de rompecabezas y otras herramientas de aprendizaje basadas en juegos en el desarrollo de materiales educativos podría aumentar la motivación de los estudiantes. Además de los métodos de enseñanza tradicionales, es esencial emplear una variedad de técnicas de enseñanza que se adapten a los diversos estilos de aprendizaje de los estudiantes. Métodos como la simulación, el aprendizaje basado en escenarios y las aulas invertidas podrían apoyar aún más los procesos de aprendizaje de los estudiantes.

Por último, se necesita más investigación para examinar los efectos de las técnicas de enseñanza, como los rompecabezas, en diferentes materias y grupos de estudiantes. De esta manera, se podrá evaluar la eficacia de los métodos de enseñanza desde una perspectiva más amplia y se podrán adoptar enfoques innovadores en la educación.

## Data Availability

Los conjuntos de datos generados y/o analizados durante el presente estudio están disponibles en el artículo.
